# Sp1 and KLF15 regulate basal transcription of the human *LRP5 *gene

**DOI:** 10.1186/1471-2156-11-12

**Published:** 2010-02-08

**Authors:** Jiangxia Li, Yang Yang, Baichun Jiang, Xiyu Zhang, Yongxin Zou, Yaoqin Gong

**Affiliations:** 1 Key Laboratory for Experimental Teratology of the Ministry of Education and Institute of Medical Genetics, Shandong University School of Medicine, Jinan, Shandong, China

## Abstract

**Background:**

LRP5, a member of the low density lipoprotein receptor superfamily, regulates diverse developmental processes in embryogenesis and maintains physiological homeostasis in adult organisms. However, how the expression of human *LRP5 *gene is regulated remains unclear.

**Results:**

In order to characterize the transcriptional regulation of human *LRP5 *gene, we cloned the 5' flanking region and evaluated its transcriptional activity in a luciferase reporter system. We demonstrated that both KLF15 and Sp1 binding sites between -72 bp and -53 bp contribute to the transcriptional activation of human *LRP5 *promoter. Chromatin immunoprecipitation assay demonstrated that the ubiquitous transcription factors KLF15 and Sp1 bind to this region. Using *Drosophila *SL2 cells, we showed that KLF15 and Sp1 trans-activated the *LRP5 *promoter in a manner dependent on the presence of Sp1-binding and KLF15-binding motifs.

**Conclusions:**

Both KLF15 and Sp1 binding sites contribute to the basal activity of human *LRP5 *promoter. This study provides the first insight into the mechanisms by which transcription of human *LRP5 *gene is regulated.

## Background

The *LRP5 *gene, located on human chromosome 11q13, contains 23 exons encoding a 1615 amino acid single-pass transmembrane receptor that belongs to the low density lipoprotein (LDL) receptor superfamily. LRP5 is highly conserved between species. There is 95% identity between human and mouse LRP5 protein[1], and 40% identity with the *Drosophila *orthologous gene, Arrow [2]. Additionally, LRP5 bears 71% amino acid identity with LRP6[3]. LRP5 contains an extracellular domain, a membrane-spanning domain, and an intracellular domain. The extracellular domain, located at the N-terminus, consists of four EGF-receptor-like cysteine-rich repeats with associated YWTD spacer domains and three LDL receptor-like ligand binding domains[1]. LRP5 is expressed in multiple adult and embryonic tissues, including bone, macrophages, fat, brain, heart, liver, skin and pancreas, with strongest expression occurring in the liver[1]. In bone, it is expressed by osteoblasts of the endosteal and trabecular bone surfaces but not by osteoclasts[4,5].

Shortly after the discovery of LRP5, multiple lines of evidence indicate that the LRP5 plays an important role in bone formation through the Wnt signaling pathway[4,6,7]. Loss-of-function mutations in human *LRP5 *gene cause the osteoporosis-pseudoglioma syndrome (OPPG), an autosomal-recessive condition of juvenile onset characterized by blindness due to aberrant vitreo-retinal vascular growth and osteoporosis resulting in fractures and deformation[4]. Gain-of-function mutation (G171V) in the same gene, on the other hand, resulted in high bone mass (HBM) [6-8]. Consistent with the observations in human, bone formation and osteoblast proliferation are decreased in *Lrp5*-deficient mice[5], and transgenic mice that expressed the *LRP5 *G171V mutation in osteoblasts exhibited enhanced osteoblastic activity, reduced osteoblast apoptosis, and a high bone mass phenotype [9]. Furthermore, independent epidemiological studies have linked sequence variants in *LRP5 *gene with differences in bone mineral density (BMD) and/or fracture risk [10-13].

Consistent with the observation with the OPPG patients, other diseases associated with abnormal angiogenesis or vascularization of the eye are also associated with mutations in *LRP5 *or its paralog *LRP6 *as well as proteins that interact with the two receptors, including familial exudative vitroretinopathy [14-16], Norrie disease[17], and macular degeneration [18]. In addition to its essential role in bone accrual and eye development, the LRP5 is also required for normal cholesterol and glucose metabolism[19,20]. Thus, LRP5 is a widely expressed receptor and is critical for normal development. It regulates diverse developmental processes in embryogenesis and maintains physiological homeostasis in adult organisms. Interestingly, the most studied functions of LRP5 are related to its roles as a co-receptor for the frizzled family of Wnt receptors. Yadav et al recently demonstrated the existence of a novel regulatory pathway whereby gut-derived 5-hydroxytryptamine indirectly mediates the function of LRP5 in skeletal differentiation [21].

Despite its important roles in several developmental processes, the transcriptional regulation of *LRP5 *remains unclear. Information regarding the regulation of *LRP5 *gene expression may help us to understand its function and find potential new anabolic targets for conditions such as osteoporosis. To this end, we cloned the 5' flanking region and evaluated its transcriptional activity in a luciferase reporter system. We demonstrate that both Krüppel-like factor 15(KLF15) and specificity protein 1(Sp1) binding motifs are essential for human *LRP5 *promoter activity.

## Results

### Identification of putative transcription factor binding sites in human *LRP5 *promoter region

**Figure 1:**
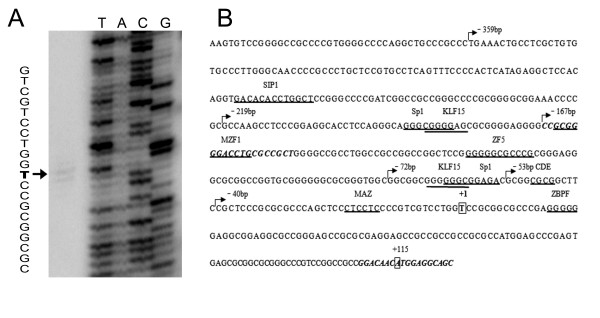
**Identification of the TSS of the *LRP5 *gene and the putative transcription factor binding sites in human *LRP5 *promoter**. (A) The transcription start site (TSS) was determined by primer extension, and the extended product is indicated by the arrow. (B) Nucleotide sequence surrounding the 5' end of human *LRP5*. The numbering of the sequence is relative to TSS. Distinct DNA binding sites for transcription factors are indicated (underlined). The numbers above the sequence referred to the 5' position of serial deletion constructs. The primers used in ChIP assay are indicated in italic and bold.

Before characterizing human *LRP5 *promoter, primer extension was performed to identify the transcription start site (TSS) of human *LRP5 *gene. We observed that the TSS of human *LRP5 *gene was located 40 nucleotides upstream of the 5' end of the published cDNA sequence (GenBank accession No: NM_002335), corresponding to a T residue at position 114 upstream of the ATG translation start site (Figure. 1A). In order to identify putative transcription factor binding sites, we analyzed the human *LRP5 *5'-flanking sequence from -2381 to +125 (relative to transcription start site) using the programs MatInspector http://www.genomatix.de/products/MatInspector/ and AliBaba 2.1 http://www.gene-regulation.com/pub/programs.html. No canonical TATA box or CAAT-like sequences were evident. However, several potential transcription factor binding motifs were identified in the promoter region close to transcription start site, including Sp1, KLF15, MZF, MAZ, CDE and ZBPF (Figure. 1B).

### The minimal promoter of human *LRP5 *gene is located within the region -72/-53 relative to the transcription start site

**Figure 2:**
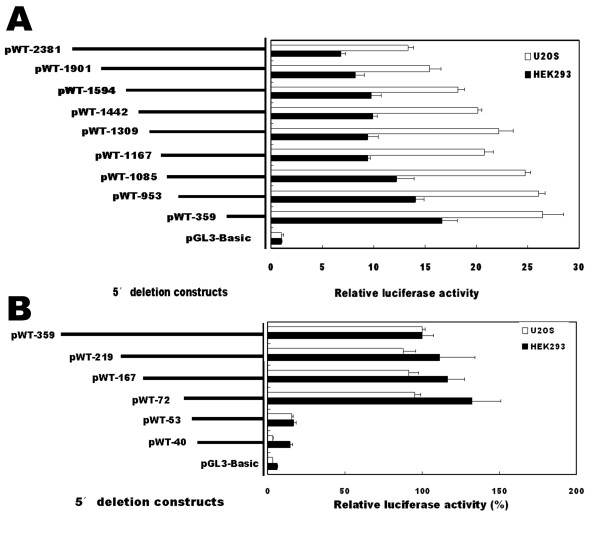
**A sequence located between -72 and -53 confers basal transcriptional activity of human *LRP5 *promoter**. (A and B) Serial 5' deletion constructs were transfected into U2OS and HEK293 cells, and the relative luciferase activity was determined. The activity of pGL3-basic construct in A or that of pWT-359 construct in B was arbitrarily set to 100%, and the relative luciferase activity of the other constructs was calculated accordingly. Each bar represents the value of mean ± SEM.

To determine the minimal region required for basal activity of the *LRP5 *promoter, a series of deletion constructs were generated, designated as pWT-2381, pWT-1901, pWT-1594, pWT-1442, pWT-1309, pWT-1167, pWT-1085, pWT-953 and pWT-359 based on their variable 5' end. U2OS and HEK293 cells, representing cells of osteoblastic and non-osteoblastic origin, respectively, were used to evaluate the promoter activities and to determine whether there exist regulatory elements that are specific to osteoblastic cells. Figure 2A summarizes the results of these transfection analyses. Maximal promoter activity was observed with the construct pWT-359 in both U2OS and HEK293 cells. When the sequence length increased up to -2381, a 50% reduction in luciferase activities was observed, suggesting the presence of negative regulatory elements between -2381 and -359. These results indicate that the human *LRP5 *proximal promoter elements are located downstream -359 and that there are essential positive control elements within the sequence from -359 to +125. Similar trends were observed in U2OS cells and HEK293 cells, suggesting that the regulatory sequences within the region examined were not cell-specific.

To further define the sequence required for proximal *LRP5 *promoter activity, a new series of deletion constructs were then generated by PCR, designed as pWT-219, pWT-167, pWT-72, pWT-53 and pWT-40. The relative luciferase activities were compared to that of pWT-359 construct which was set as 100%. As shown in figure 2B, no significant reduction of the reporter activity was detected in the HEK293 and U2OS cell lines when deletions were made between -359 and -72. However, deletion from nucleotides -72 to -53 resulted in 80% reduction in the promoter activity. These deletion experiments indicate that the essential positive control elements for *LRP5 *promoter activity are located between -72 and -53. Therefore, we decided to firstly focus our interest on the minimal promoter of human *LRP5 *gene.

### The KLF15 and Sp1 binding sites between -72 and -53 contribute to human *LRP5 *promoter activity

**Figure 3:**
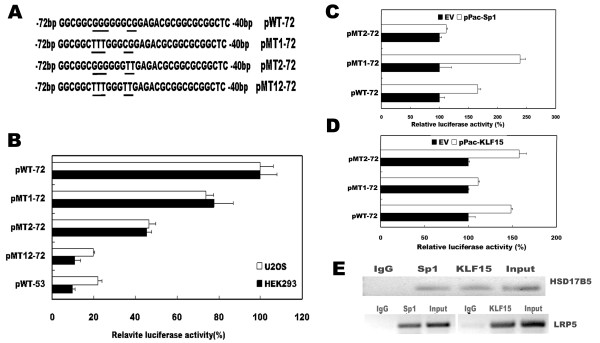
**KLF15 and Sp1 binding sites located between -72 and -53 contribute to the basal transcriptional activity of human *LRP5 *promoter**. (A) Schematic representation of the KLF15 and Sp1 elements. Point mutations (underlined) were introduced to change the binding sites. (B) Constructs with native (pWT-72) or mutant KLF15 and/or Sp1 sites were transfected into HEK293 and U2OS cells, and the luciferase activity was determined. The luciferase activity of pWT-72 was arbitrarily set to 100%, and the activities of other constructs were calculated accordingly. (C and D) Sp1 and KLF15 transactivated the *LRP5 *promoter only if Sp1 and KLF15 binding sites were present. SL2 cells were cotransfected with either wild type construct (pWT-72) or mutated constructs (pMT1-72 or pMT2-72) along with the Sp1 (3C) or KLF15 (3D) expression constructs, and the relative luciferase activity was determined. The luciferase activity cotransfected with control vector (empty vector, EV) was set to 100%, and the relative activity under KLF15 or Sp1 stimulation was calculated accordingly. (E) Chromatin immunoprecipitation assay of KLF15 and Sp1 binding to human *LRP5 *promoter. The bindings of KLF15 and Sp1 to the human *HSD17B5 *promoter were used as a positive control (upper panel). The bindings of KLF15 to the *LRP5 *promoter (lower right panel) and the binding of Sp1 to the *LRP5 *promoter (lower left panel) were determined by ChIP using anti-KLF15 and anti-Sp1 antibodies, respectively. Anti-IgG antibodies were used as a negative control. The associated chromatin DNA fragments were amplified with the primer pairs flanking the Sp1 and KLF15 binding sites. Chromatin DNA input as described in the material and methods was subjected to the same PCR amplification.

The region between -72 and -53 is a GC-rich region, containing two overlapped transcription factor binding sites, one for KLF15 and the other for Sp1. To determine the contribution of the Sp1 and/or KLF15 sites to the promoter activity, point mutations were introduced into construct pWT-72 by PCR-based mutagenesis to generate either single or double mutant reporter plasmids which were designated as pMT1-72, pMT2-72, and pMT12-72, respectively (Figure. 3A). In pMT1-72, the KLF15 motif GCGGGGGG was mutated to GC**TTT**GGG, and in pMT2-72 the Sp1 motif GGGCGGA was mutated to GGG**TT**GA. The double mutant construct pMT12-72 contained mutations in both motifs. The luciferase activities driven by the native (pWT-72) and mutated constructs were measured in HEK293 and U2OS cells. As illustrated in figure 3B, relative to pWT-72, pMT1-72 and pMT2-72 showed 22-25% and 53-55% reduction in promoter activity, respectively, confirming the positive regulatory role of the KLF15 site and the Sp1 site. The luciferase activity driven by double mutant (pMT12-72) was reduced to 11% in HEK293 and 20% in U2OS cells, the values as observed in the construct that lacks both KLF15 and Sp1 binding sites (pWT-53). These results indicate that both KLF15 and Sp1 binding sites between -72 and -53 of human *LRP5 *promoter contribute to the basal activity of human *LRP5 *transcription.

### Trans-activation of human *LRP5 *promoter by KLF15 and Sp1

To determine whether KLF15 and Sp1 functionally modulate the *LRP5 *promoter activity, *Drosophila *SL2 cells, which are deficient in Sp1 and KLF15, were utilized. SL2 cells were cotransfected with either the wild type pWT-72 construct or the constructs with mutated Sp1 and/or KLF15 binding sites along with the KLF15 or Sp1 expression plasmids. As shown in figure 3C and 3D, Sp1 and KLF15 stimulated *LRP5 *promoter activity of the wild type construct. However, the mutant constructs were not activated by co-transfection with corresponding expression constructs, pPacSp1 (Figure 3C) or pPacKLF15 (Figure 3D), indicating that transactivation of the *LRP5 *by Sp1 and KLF15 depends on the presence of Sp1-binding and KLF15-binding motifs in the *LRP5 *promoter.

### In vivo binding of Sp1 and KLF15 to human *LRP5 *promoter

To verify in vivo promoter binding by transcription factors KLF15 and Sp1, chromatin immunoprecipitation (ChIP) was performed on HEK293 cells. The sonicated nuclear extract was subjected to immunoprecipitation with anti-KLF15 and anti-Sp1 antibodies, and the associated chromatin DNA fragments were amplified with the primer pairs flanking the KLF15 and Sp1 binding sites as shown in figure 1B. As a negative control, a reaction was included with antibodies against IgG. The *HSD17B5 *promoter which contains both Sp1 and KLF15 binding sites was used as a positive control [22]. As shown in figure 3E, Anti-KLF15 and anit-Sp1 antibodies, but not IgG antibodies, could immunoprecipitate the *HSD17B5 *promoter and *LRP5 *promoter in HEK293 cells. These results confirm that human *LRP5 *promoter is indeed bound by Sp1 and KLF15 transcription factors in vivo.

## Discussion

In the present study, we have for the first time cloned the human *LRP5 *promoter and analyzed its transcriptional regulation. We demonstrated that both KLF15 and Sp1 binding sites located between -72 and -53 contribute to basal promoter activity of human *LRP5 *gene. There are several lines of evidences. First, by generating a series of 5' deletions, we show that the core promoter is located between nucleotides -72 and -53 relative to transcription start site. Second, changing the KLF15 and Sp1 motifs within this region by introducing point mutations strongly reduced *LRP5 *promoter activity. Third, in *Drosophila *SL2 cells that lack endogenous Sp1 and KLF15, we show that expression of KLF15 and Sp1 is able to trans-activate the *LRP5 *promoter that contains Sp1-binding and KLF15-binding motifs. Finally, ChIP assay confirmed the binding of KLF15 and Sp1 to the *LRP5 *promoter in vivo.

Kruppel-like factors (KLFs), a subclass of the zinc-finger family of transcriptional regulators, are critical regulators of growth and differentiation in a broad range of mammalian cell types[23,24]. Members of this gene family have been characterized as regulators of both tissue-specific and ubiquitous genes and can function as transcriptional activators and/or repressors depending on promoter context[24,25]. KLF15 is expressed in multiple tissues, including liver, white and brown adipose tissues, kidney, heart, and skeletal muscle, with strongest expression occurring in the liver and kidney[26]. KLF15 appears to be bi-functional, as it represses the *Rho *and *IRBP *promoters [27] and the kidney-specific *CLC-K1 *and *CLC-K2 *promoters [28], but activates the *GLUT4 *promoter[26] and *AceCs2 *promoter[29]. In this study, mutation of the KLF15 binding motif lead to a reduction of the promoter activity in HEK293 and U2OS cells, indicating that this motif located between -72 and -53 functions as a positive regulatory element and activates the human *LRP5 *promoter.

The zinc-finger protein Sp1, which belongs to Sp family, was identified as the major protein forming complexes with oligonucleotides containing Sp1 and KLF15 binding motifs. The Sp family has 9 members containing three conserved zinc finger DNA binding domains, designated Sp1 to Sp9. Among them, Sp1, Sp3 and Sp4 are closely related containing two major glutamine-rich transactivation domains that are essential for transcription activation. They bind GC motifs to regulate the expression of housekeeping, tissue-specific and viral genes[30,31]. In the present study, we identified a functional Sp1 binding site within the core promoter of human *LRP5 *gene. Mutation of the Sp1 site decreased the promoter activity by 53% in U2OS cells and 55% in HEK293 cells. Expression of Sp1 stimulated *LRP5 *promoter activity in a Sp1-binding site dependent manner. Therefore, Sp1 binding site located between -72 and -53 contributes to activation of the *LRP5 *promoter.

KLF15 can directly interact with MEF2, and activates the *GLUT4 *promoter in a synergetic manner[26]. Yamamoto et al demonstrated that KLF15 in combination with Sp1, synergistically activates the *AceCS2 *promoter[29]. In human *LRP5 *promoter, KLF15 and Sp1 motifs overlap. Simultaneously mutating KLF15 and Sp1 binding elements decreased the promoter activity more dramatically. This activation may be mediated by a synergetic interaction between the KLF15 and Sp1 factors resulting in a combined effect on the promoter activity.

## Conclusions

Based on these data, we conclude that both KLF15 and Sp1 binding sites located between positions -72 to -53 relative to the transcription start site play critical roles in regulating the basal transcription of human *LRP5 *gene. The characterization of the human *LRP5 *basal promoter represents a starting point to further unravel the underlying molecular mechanisms that regulate human *LRP5 *expression, contributing to the understanding of how the function of LRP5 is regulated during bone formation and eye development.

## Methods

### Cell culture

Human osteosarcoma cells (U2OS) and human embryonic kidney cells (HEK293) were purchased from the American Type Culture Collection (Manassas, VA). U2OS Cells were cultured in McCoy's 5A medium (Gibco, Invitrogen). HEK293 cells were maintained in Dulbecco's modified Eagle's medium (DMEM) (Gibco, Invitrogen). All media contained 10% fetal bovine serum (FBS), 100 units/ml penicillin, and 100 mg/ml streptomycin (Gibco BRL). Cells were incubated in a humidified incubator equilibrated with 5% CO_2 _at 37°C. *Drosophila melanogaster *Schneider cell line 2 (SL2; ATCC No. CRL-1963) was purchased from Invitogen (Carlsbad, CA) and maintained in Schneider's insect medium supplemented with 10% fetal bovine serum and 2 mM L-glutamine plus 100 units/ml penicillin and100 mg/ml streptomycin, and cultured at 25°C.

### RNA isolation and primer extension

When reaching 80% confluence in 75 cm^2 ^flasks, U2OS cells were washed twice with ice-cold PBS and total RNA was isolated with the use of TRIzol reagents (Sigma-Aldrich, St. Louis, USA) according to the manufacturer's protocol. Any potential DNA contamination was removed by RNase-free DNase treatment. The transcription start site of human *LRP5 *gene was determined by primer extension using a primer extension system (Promega). An antisense primer (5'-GGCGGCGCTGCCTCCATGTTGTCCG-3'), corresponding to positions +107 to +131 (relative to transcription start site) of human *LRP5 *cDNA (GenBank accession No. NM_002335), was 5'end-labeled with [γ -^32^P] ATP (3000 Ci/mmol) using T_4 _polynucleotide kinase. The ^32^P-labeled primer of 10^6 ^cpm was added to 50 μg of total RNA from U2OS cells in a final volume of 20 μl. The reverse transcription was performed with AMV reverse transcriptase according to the manufacturer's protocol. The reaction products were analyzed on 6% polyacrylamide/7 M urea gels alongside manual sequencing reaction of the plasmid DNA containing the 5'-flanking region of human *LRP5 *gene with the same end-labeled primer.

### Plasmids and Constructs

Utilizing the NCBI GenBank, we identified the genomic sequence of human *LRP5 *and found a BAC clone (PR11-149G19) containing the human *LRP5 *gene. We therefore amplified the proximal 5' region of human *LRP5 *(-2381 to +125 bp relative to transcription start site) by PCR using PR11-149G19 clone as the template. The primers (LRP5F and LRP5R) used are listed in Table 1. PCR product was cloned into pMD18-T vector (TaKaRa), and then subcloned into the firefly luciferase reporter vector pGL3-basic, a promoter-less luciferase expression plasmid (Promega, Madison, WI), creating pWT-2381. This construct was subsequently used as a template for the generation of a series of 5' unidirectional truncation mutants by PCR using assorted sense primers with a common antisense primer, LRP5R/HindIII (Table 1).

**Table 1:** The oligonucleotide sequence of primers used in PCR

Name	Sequence(5'→3')	Strand	Location
LRP5F	GCCTGACTGAGGAGCTGAAG	sense	-2381 ~ -2361
LRP5R	GCTGCCTCCATGTTGTCC	antisense	**+**105 ~ +125
LRP5R/HindIII	CCC**AAGCTT**GCTGCCTCCATGTTGTCC	antisense	+105 ~ +125
-1901 F/KpnI	GAC**GGTACC**AGCTACGATCACACCACTAC	Sense	-1901 ~ -1881
-1594 F/KpnI	GAC**GGTACC**GGCTGCCATGAGATCAGGTG	Sense	-1594 ~ -1574
-1442 F/KpnI	GAC**GGTACC**AGGCTGTGCTGGTAGAGCTG	sense	-1442 ~ -1422
-1309 F/KpnI	GAC**GGTACC**CTGTACCTTCTACCACCAGC	Sense	-1309 ~ -1279
-1167 F/KpnI	GAC**GGTACC**TTGGTAGCAGCTCAAGTGTC	Sense	-1167 ~ -1147
-1085 F/KpnI	GAC**GGTACC**TTCTTCATGGCCTGTCAATG	Sense	-1085 ~ -1065
-953 F/KpnI	GAC**GGTACC**AATCTGGAGGAACACACACG	Sense	-953 ~ -933
-359 F/KpnI	GAC**GGTACC**TGAAACTGCCTCGCTGTGTG	Sense	-359 ~ -339
-219 F/KpnI	GAC**GGTACC**GCCAAGCCTCCCGGAGGCAC	Sense	-219 ~ -199
-167 F/KpnI	GAC**GGTACC**CCGCGGGGACCTGCGCCGCT	Sense	-167 ~ -147
-72 F/MluI	CG**ACGCGT**GGCGGCGGGGGGCGGAGAAG	Sense	-72 ~ -52
-53 F/MluI	CG**ACGCGT**GCGGCGCGGCTTCCGCTCCCG	Sense	-53 ~ -33
-40 F/KpnI	GAC**GGTACC**CGCTCCCGCGCGCCCAGCTC	Sense	-40 ~ -20
-72MT1/MluI	CG**ACGCGT**GGCGGC***TTT***GGGCGGAGAAG	Sense	-72 ~ -52
-72MT2/MluI	CG**ACGCGT**GGCGGCGGGGGG***TT***GAGAAGCGGCG	Sense	-72 ~ -52
-72MT12/MluI	CG**ACGCGT**GGCGGC***TTT***GGG***TT***GAGAAGCGGCG	Sense	-72 ~ -52

Location: Nucleotide positions relative to the transcription start site (+1) of the human *LRP5 *gene.

Restriction sites are indicated in bold. The nucleotides of specific mutagenesis are indicated in italic and bold.

Mutant versions for specific binding sites were prepared by PCR-based mutagenesis from pWT-72, using the common reverse primer LRP5R/Hind III combining with the forward primers -72MT1/MluI, -72MT2/MluI and -72MT12/MluI, respectively (Table 1). The PCR conditions were as follows: the samples were denatured at 94°C for 4 min, and then 30 cycles were applied: 94°C for 40 s, 60°C for 40 s and 72°C for 50 s, the samples were further held at 72°C for 10 min. PCR products were digested with KpnI (or MluI) and HindIII enzymes, gel-purified, and ligated to the pGL3-basic vector. The sequences of all constructs were confirmed by restriction enzyme digestion and direct sequencing.

pPac, a *Drosophila *actin 5C promoter-driven expression vector, pPac-gal containing an *Escherichia coli*-galactosidase, and pPacSp1 containing Sp1, were provided by Dr. G. Suske (Philipps-Universität Marburg, Germany)[32]. The KLF15 coding cDNA was generated by reverse transcription-PCR from HEK293 total RNA with forward (5'-**CGCGGATCC**ATGGTGGACCACTTACTTCCAGTGGACGAG-3') and reverse (5'- **CCGCTCGAG**TCAGTTCACGGAGCGCACGGAGCGGCTGCTC-3') primers. BamHI and XhoI restriction sites, respectively (bold), were introduced into the primers for subcloning. The amplicons were digested with BamHI and XhoI and inserted into the *Drosophila *pPac expression vector after excision of the Sp1 coding sequence from pPacSp1. Construct sequences were confirmed by DNA sequencing.

### Transient transfection and luciferase assay

Transient transfections were performed with the use of Lipofectamine 2000 reagent (Invitrogen) as previously described[33]. Briefly, U2OS and HEK293 cells were seeded into 48-well plates at a density of 5 × 10^4 ^cells per well and 4 × 10^4 ^cells per well, respectively, the day before transfection. For each well of cells, 0.4 μg of the *LRP5 *promoter constructs (deletion and site-directed mutants) were co-transfected with 0.02 μg of pRL-TK plasmid (Promega). The cells were cultured for 24 hours after transfection and then harvested. Luciferase activity was measured using the Dual-Luciferase Reporter Assay System (Promega). To normalize for transfection efficiency, the promoter activity of each construct was expressed as the ratio of firefly luciferase activity to *Renilla *luciferase activity. For each construct, more than three independent experiments with U2OS and HEK293 cells were performed in triplicate.

For transient transfection of SL2 cells, one day prior to transfection, cells were plated into 24-well plates at a density of 5 × 10^5 ^cells/well. Cells were transfected with 0.5 μg of indicated reporter construct (pWT-72, pMT1-72 and pMT2-72) and 0.1 μg of pPacSp1 or pPac KLF15 expression vector or empty vector pPac0 together with pPac-β-gal vector (internal reference encoding the β-galactosidase gene). Twenty-four hours after transfection, the cells were harvested and luciferase activity was measured using Bright-Glo luciferase assay system (Promega) and β-galactosidase activity was measured using Beta-Glo assay system (Promega). Luciferase activities were normalized to β-galactosidase activity, reported as means of three independent experiments, and each performed in duplication.

### Chromatin immunoprecipitation assays (ChIP)

ChIP assays were performed using an EZ-ChIP assay kit (Upstate Biotechnologies, Millipore, MA) according to the manufacturer's instructions. Briefly, HEK293 cells were treated with formaldehyde and lysed with lysis buffer. The cell lysates were sonicated to generate 200-1000 bp DNA fragments, and cross-linked proteins were immunoprecipitated by incubation with antibodies against Sp1 (Santa Cruz, CA), KLF15 (Abcam, cambridge, UK), and IgG, respectively. A DNA sample from sonicated nuclear lysates that underwent reverse cross-link and phenol/chloroform extraction was used as positive control (input). A DNA sample immunoprecipitated by antibodies against IgG was used as negative control. Immunoprecipitated DNA was detected by PCR amplification using primer LRP5-ChIP-forward (5'-CCGCGGGGACCTGCGCCGCT-3') combining with primer LRP5R. As a positive control, specific primers of *HSD17B5 *promoter (5'-TTCTCCACAGACCATATAAG-3' and 5'-TTCCCTGTCACTTGTCTGACT-3') were designed to amplify the promoter region containing Sp1 the KLF15 binding sites [22]. PCR products were separated on 2% agarose gel and visualized by ethidium bromide staining.

### Statistical analysis

The statistical significance of differences between experimental groups was calculated using the Student's test. Group differences were considered significant if P < 0.05.

## List of abbreviations

LRP5: low-density lipoprotein receptor related protein; Sp1: specificity protein 1; KLF15: Krüppel-like factor 15; ChIP: chromatin immunoprecipitation; HSD17B5: human 17-hydroxysteroid dehydrogenase type 5 gene.

## Authors' contributions

JL performed luciferase reporter assay and ChIP analysis, and drafted the manuscript. YY and BJ participated in the primer extension and reporter assays. XZ and YZ assisted in RNA isolation and vector construction. YG contributed to the conception and the design of the study and the writing of the manuscript. All authors read and approved the final manuscript.
